# Non-invasive visualization of tumor infiltrating lymphocytes in patients with metastatic melanoma undergoing immune checkpoint inhibitor therapy: a pilot study

**DOI:** 10.18632/oncotarget.25666

**Published:** 2018-07-13

**Authors:** Svetomir N. Markovic, Filippo Galli, Vera J. Suman, Wendy K. Nevala, Andrew M. Paulsen, Joseph C. Hung, Denise N. Gansen, Lori A. Erickson, Paolo Marchetti, Gregory A. Wiseman, Alberto Signore

**Affiliations:** ^1^ Department of Oncology, Mayo Clinic, Rochester, MN, USA; ^2^ Nuclear Medicine Unit, Department of Medical-Surgical Sciences and of Translational Medicine, “Sapienza” University of Rome, Rome, Italy; ^3^ Department of Health Sciences Research, Mayo Clinic, Rochester, MN, USA; ^4^ Department of Immunology, Mayo Clinic, Rochester, MN, USA; ^5^ Department of Radiology, Division of Nuclear Medicine, Mayo Clinic, Rochester, MN, USA; ^6^ Department of Laboratory Medicine and Pathology, Mayo Clinic, Rochester, MN, USA; ^7^ Oncology Unit, Department of Clinical and Molecular Medicine, “Sapienza” University of Rome, and IDI-IRCCS, Rome, Italy

**Keywords:** check point inhibitors, ipilimumab, pembrolizumab, melanoma, ^99m^Tc-IL2

## Abstract

Early in the course of immunotherapy there is frequently a transient enlargement of tumor masses (pseudo-progression) due to tumor infiltration by TILs. Current clinical imaging modalities are not able to distinguished pseudo-progression from true tumor progression. Thus, patients often remain on treatment 4-8 weeks longer to confirm disease progression. Nuclear medicine offers the possibility to image immune cells and potentially discriminate pseudo-progression and progression.

We conducted a pilot study in patients with metastatic melanoma receiving ipilimumab (IPI) or pembrolizumab (PEMBRO) to assess safety and feasibility of SPECT/CT imaging with ^99m^Tc- interleukin-2 (^99m^Tc-HYNIC-IL2) to detect TILs and distinguish between true progression from pseudo- progression. Scans were performed prior to and after 12w treatment. After labelling,^99m^Tc-HYNIC-IL2 was purified and diluted in 10 mL of 5% glucose with 0.1% human serum albumin.

Of the 5 patients (2 treated with IPI and 3 with PEMBRO) enrolled, two failed to complete the second scan as they discontinued IPI due grade 3 colitis (1 patient) or patient refusal after developing multiple toxicities attributed to IPI (1 patient). Following the first scan, one patient reported to have a grade 1 pruritus with grade 1 pain. No other toxicities attributed to the radiopharmaceutical infusion were reported. Metastatic lesions could be visualized by ^99m^Tc-IL2 imaging and there was positive correlation between size and ^99m^Tc-HYNIC-IL2 uptake, both before and after 12 weeks of therapy.

The results of this pilot study demonstrate the safety and feasibility of ^99m^Tc-IL2 imaging and has led to a number of hypotheses to be tested in future studies.

## INTRODUCTION

Increased understanding of the immunobiology of cancers has led to development of immune checkpoint inhibitors (anti-CTLA4 and/or anti-PD1) such as ipilimumab, pembrolizumab, and nivolumab in advanced melanoma. These agents block ligand engagement by normal T cell down regulatory receptors (CTLA4 or PD1) thereby promoting T cell survival, most notably in the PDL1 rich tumor microenvironment. Tumor infiltration by cytotoxic T-cells, the common mechanism of action of all immune checkpoint inhibitors, may lead to transient tumor enlargement. There have been reports of a number of patients treated with these agents that experience tumor burden increases early in their treatment course that met RECIST criteria for disease progression but with continued treatment have reductions in disease burden. In clinical practice, this problem (referred to as pseudo-progression) is often handled by continuing treatment for 4–8 weeks to verify persistence of tumor enlargement, in the hopes of avoiding discontinuation of immunotherapy in patients that may be experiencing pseudo- progression and could ultimately benefit from treatment. Unfortunately, this approach can also prolong therapy with an ineffective agent. Alternative imaging approaches are being explored to provide a means to distinguish between pseudo-progression and true disease progression.

We have developed methodology for clinical visualization of T lymphocyte organ infiltration using radioactively labeled clinical grade recombinant interleukin-2 (Aldesleukin^®^, IL-2) [2]. We first began with iodine-123 (123I) labeled IL2 and demonstrated its ability to visualize lymphocyte infiltration in bowel (Celiac disease) [3–6]. Due to concerns about potential adverse effects on the thyroid from a radioactive iodine based radiopharmaceutical, the investigators next employed a 99mTechnicium labeled interleukin-2 (^99m^Tc-IL2). We have demonstrated that this approach was able to visualize lymphocyte infiltration in the bowel (Crohn's disease) as well as in the thyroid (autoimmune thyroiditis) [7–9]. Next, we examined 21 early stage melanoma primary skin lesions prior to surgical resection with ^99m^Tc-IL2 imaging for the presence of TIL and corresponding ^99m^Tc-IL2 uptake by histologic evaluation of the surgical specimen. All 21 melanoma lesions had lymphocytic infiltration at histology and 15 were judged to have positive ^99m^Tc-IL2 scan findings. Of the 17 lesions assessed for CD25 expression (IL-2 receptor), 2 lesions had no CD25+ TIL and no ^99m^Tc-IL2 uptake on imaging; 7 of the 8 lesions with CD25+ TIL constituting > 15% of all TIL had ^99m^Tc-IL2 uptake on imaging, and 4 of the 7 lesions with CD25+ TIL present at ≤ 15% of all TIL had ^99m^Tc-IL2 uptake on imaging. ^99m^Tc-IL2 estimates of TIL correlated significantly with histologic estimates of CD25+ TIL (*r* = 0.745, *p* = 0.001). Although these results were promising, the cumbersome multi-step labeling process requiring post- labeling purification and the associated cost of imaging greatly limited further clinical testing of this technology.

In recent years, we simplified the ^99m^Tc-IL2 labeling method using HYNIC-NHS and tricine as co-ligands (^99m^Tc-HYNIC-IL-2) [10]. The approach was shown to be an improvement over prior IL2 labeling efforts in terms of reduced cost, simplified production methodology, enhanced labeling reproducibility, high specific activity and high stability and capable of binding *in vitro* to CD25+ cells [10]. Moreover, bio-distribution studies in a healthy volunteer demonstrated similar results to those of prior radiopharmaceuticals with no significant toxicity [10]. Liver and the kidneys are the organs that normally metabolize IL2. ^99m^Tc-HYNIC-NHS demonstrated rapid plasma clearance with kidneys as the major organ of accumulation (peak at 1 hour post injection). Liver and spleen were also detectable, with no evidence of other organ accumulation up to 3 hours post injection.

Herein we present the results of a pilot clinical trial designed to examine the safety and feasibility of ^99m^Tc-HYNIC-IL2 SPECT/CT imaging and to examine the changes in TIL content of sites of metastatic melanoma in patients undergoing therapy with immune checkpoint inhibitors. These data will inform the development of studies to assess the ability of ^99m^Tc-HYNIC-IL2 SPECT/CT imaging to distinguish true progression from pseudo-progression,

## RESULTS

### Study cohort

Enrolment to this pilot study began March 2014 and closed May 2015 after 5 individuals were accrued due to funding limitations. None of these 5 individuals were found to be ineligible or withdrew consent prior to first ^99m^Tc-HYNIC-IL2 scan. Patient and disease characteristics are presented in

Table 1. Two patients chose to receive ipilimumab and the remaining 3 patients chose to receive pembrolizumab.

**Table 1:** Patient characteristics

	Patients (%)
Median Age 66 (range 51–82)	
Males	4 (80.0%)
ECOG performance status	
0	4 (80.0%)
1	1 (20.0%)
M stage	
M1a-b	3 (60.0%)
M1c	2 (40.0%)
Immunotherapy regimen	
Ipilimumab	2 (40.0%)
Pembrolizumab	3 (60.0%)

All 5 patients had a pre-treatment ^99m^Tc-HYNIC-IL2 scan of sites of disease involvement, namely soft tissue (2 patients.), lung (2 patients), and both soft tissue and lung (1 patient). Two patients refused to complete a ^99m^Tc-HYNIC-IL2 scan after 12 weeks of treatment. The first of these two patients was receiving pembrolizumab and was feeling quite poorly as a result of grade 3 skin rash with grade 2 fatigue, pruritus, renal insufficiency and lung infection (all attributed to pembrolizumab). The second patient discontinued ipilimumab treatment as a result of developing grade 3 colitis (attributed to ipilimumab). Following the first scan, one patient reported to have a grade 1 pruritus with grade 1 pain. No other adverse events were reported following the first or second 99mTc- HYNIC-IL2 scan. Thus, none of these 5 patients developed ^99m^Tc-HYNIC-IL2 scintigraphy limiting toxicities (0%: 90% CI: 0–45.1%)

### Imaging results

**Figure 1:**
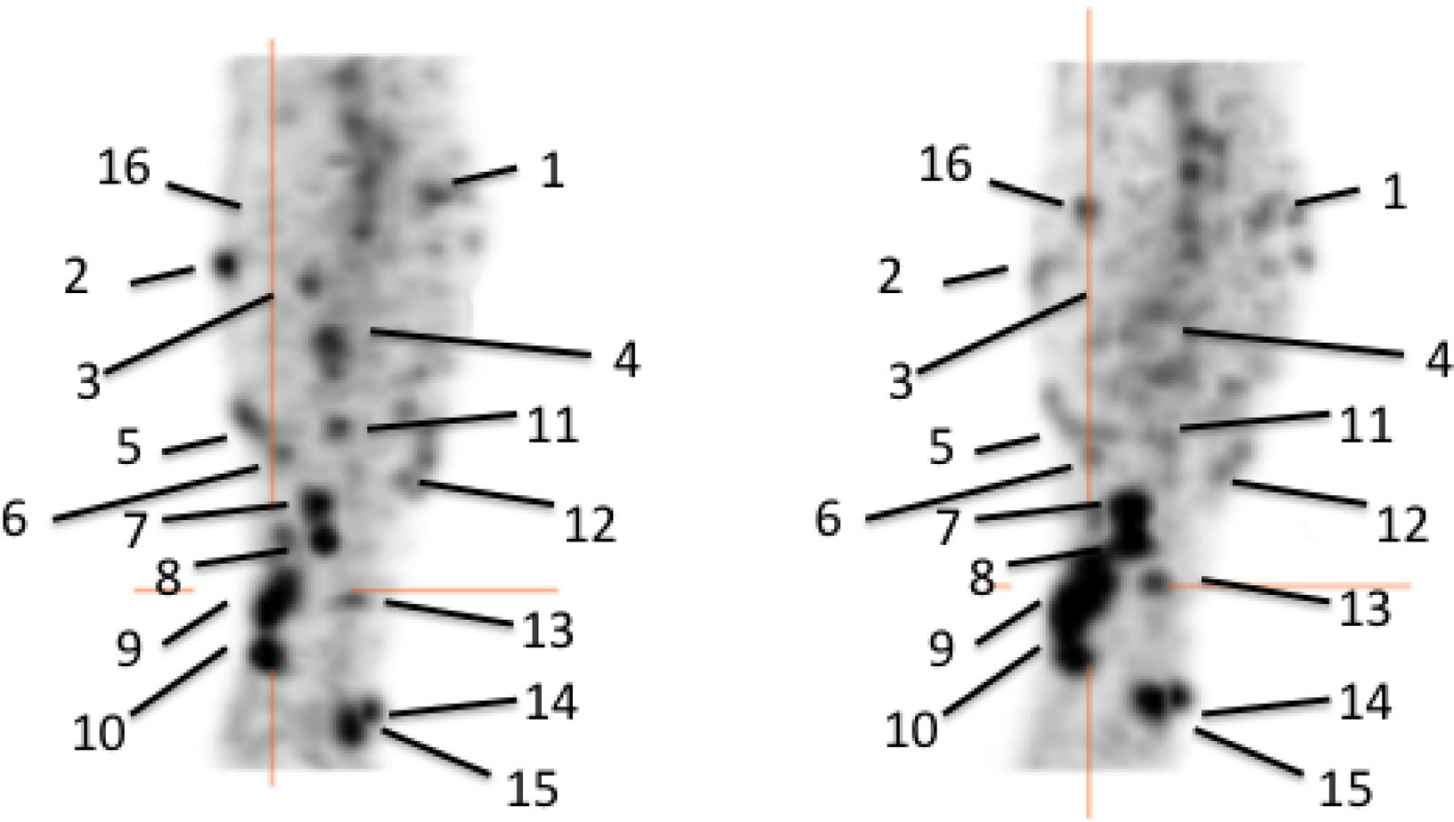
Pre-therapy (left) and post-therapy (right) images of 99mTc-HYNIC-IL2 uptake in metastatic lesions of patient #1 We identified 16 lesions in the affected leg for quantitative analysis. It can be seen that some lesion increased uptake over time, other decreased.

**Figure 2:**
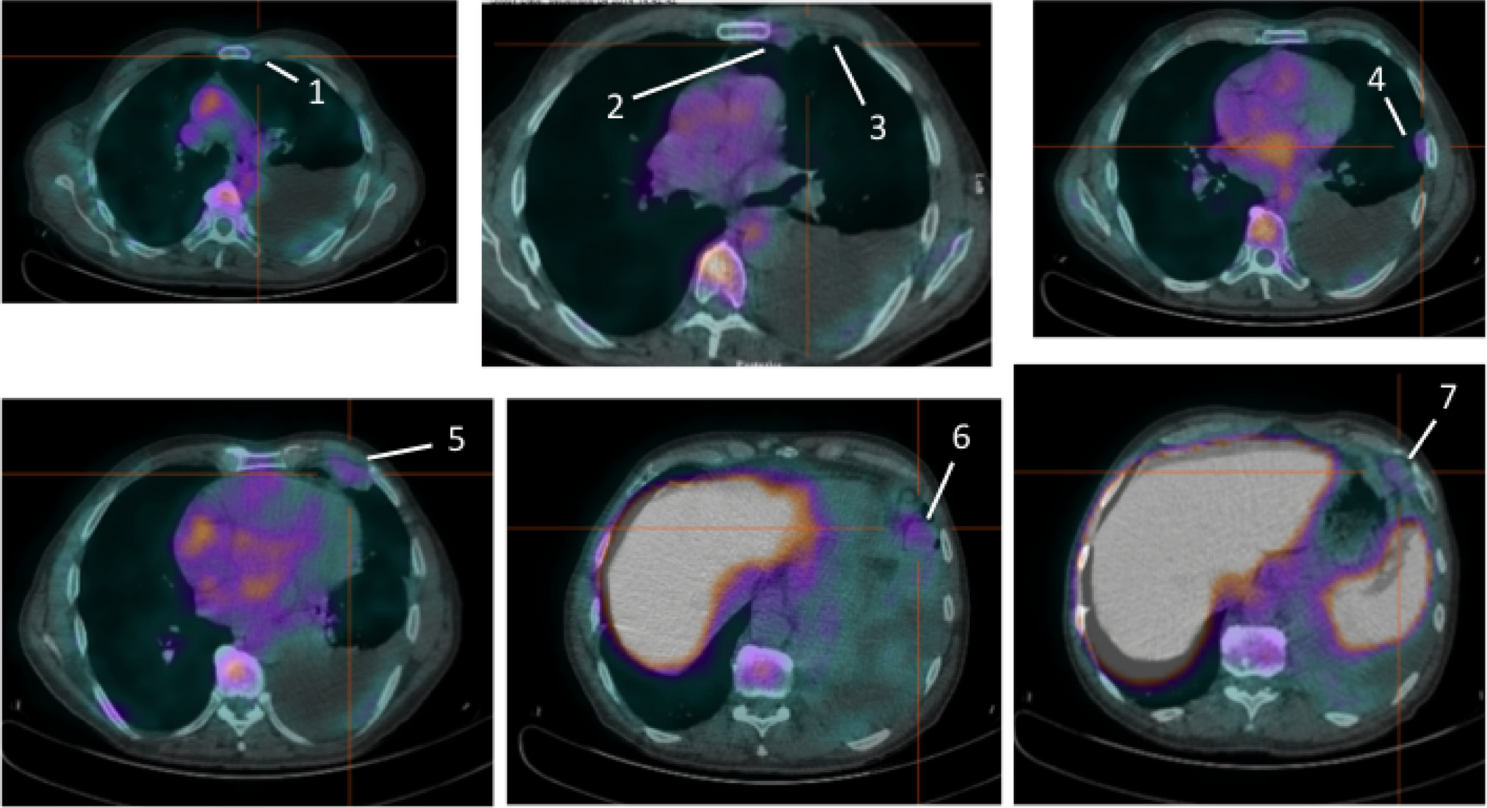
Pre-therapy images of 99mTc-HYNIC-IL2 uptake in metastatic lesions of patient #2 We identified 7 lesions in the lungs.

**Figure 3:**
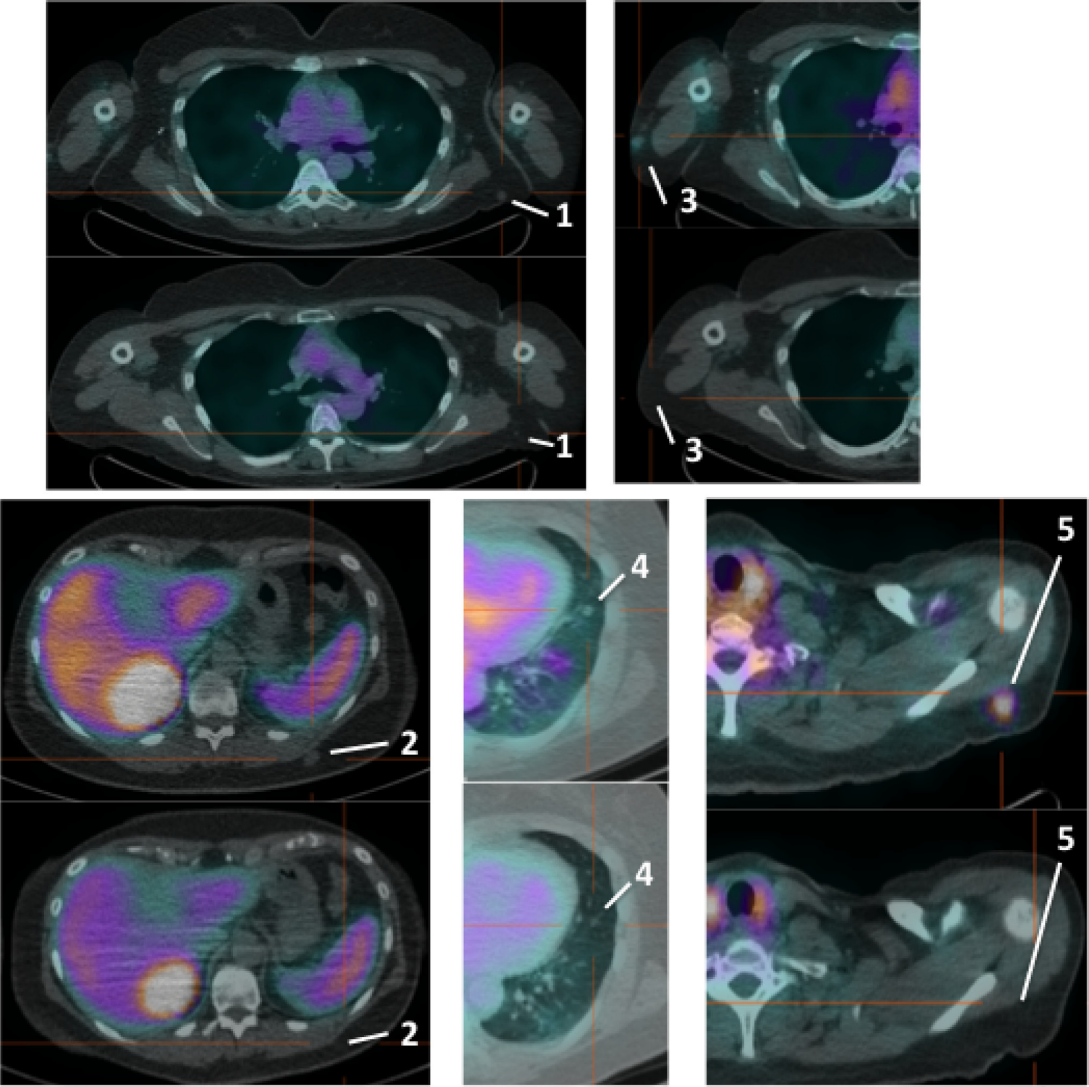
Pre-therapy (upper images) and post-therapy (lower images) images of 99mTc-HYNIC-IL2 uptake in metastatic lesions of patient #3 We identified 5 lesions in the lungs and sub-cutaneous tissue for quantitative analysis. It can be seen that most lesions disappear after therapy.

**Figure 4:**
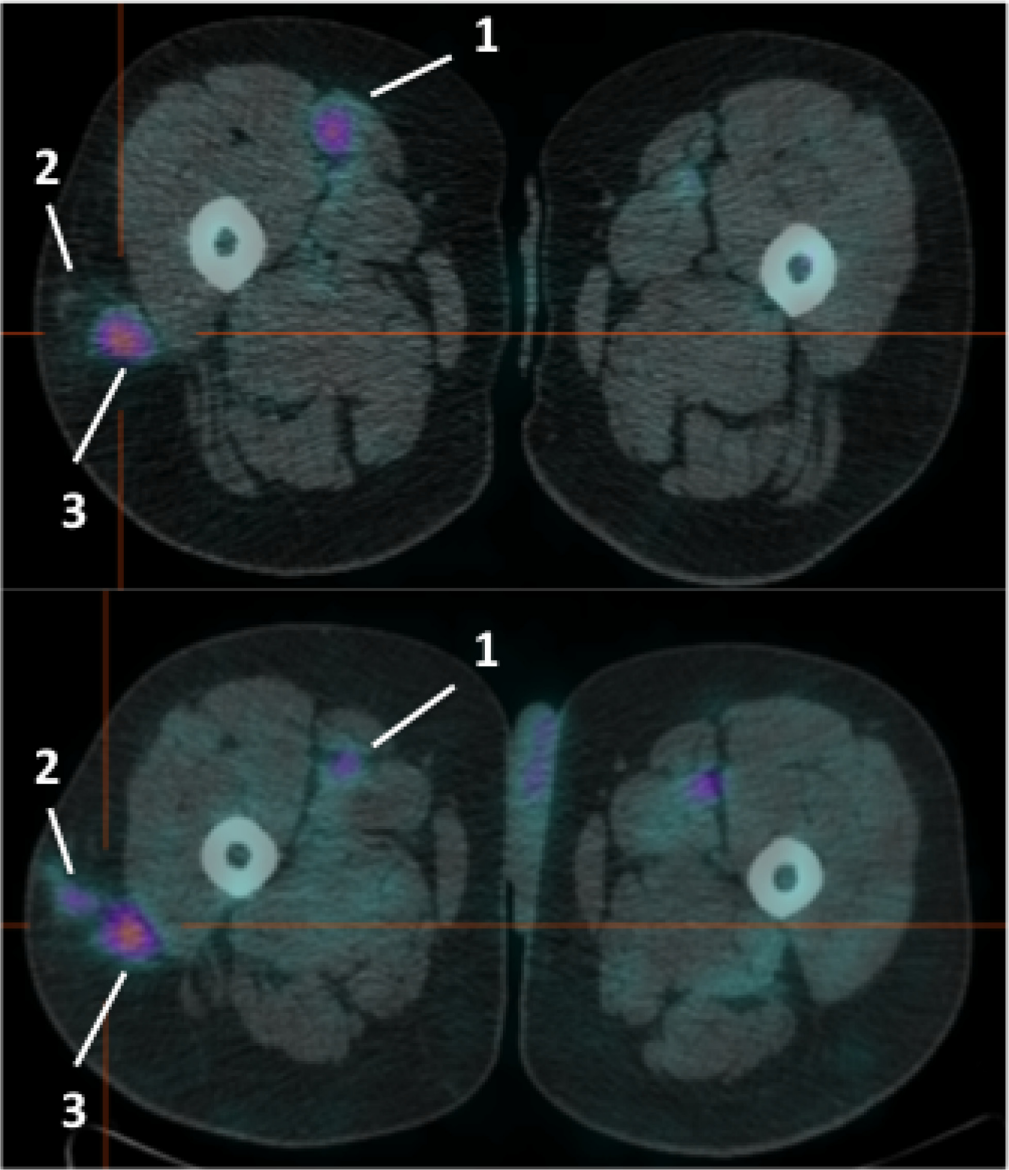
Pre-therapy (upper image) and post-therapy (lower image) images of 99mTc-HYNIC-IL2 uptake in metastatic lesions of patient #4 We identified 3 lesions in the affected leg for quantitative analysis. It can be seen that one lesion increased uptake over time, the other two decreased.

**Figure 5:**
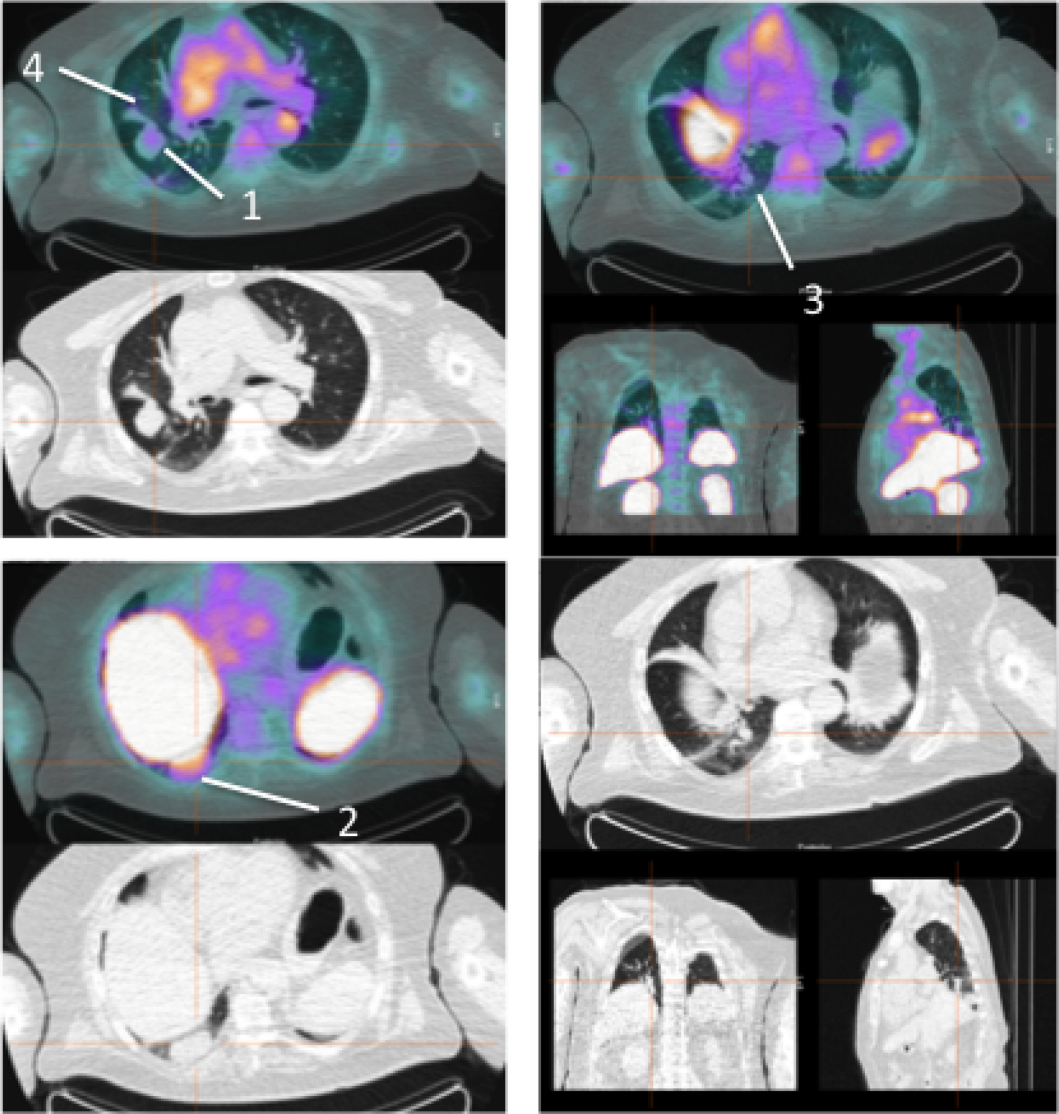
Pre-therapy images of ^99m^Tc-HYNIC-IL2 uptake in metastatic lesions of patient #5 We identified 3 lesions in the lungs. Patient refused to perform the post-therapy scan and therefore these lesions were not included for quantitative analysis.

**Figure 6:**
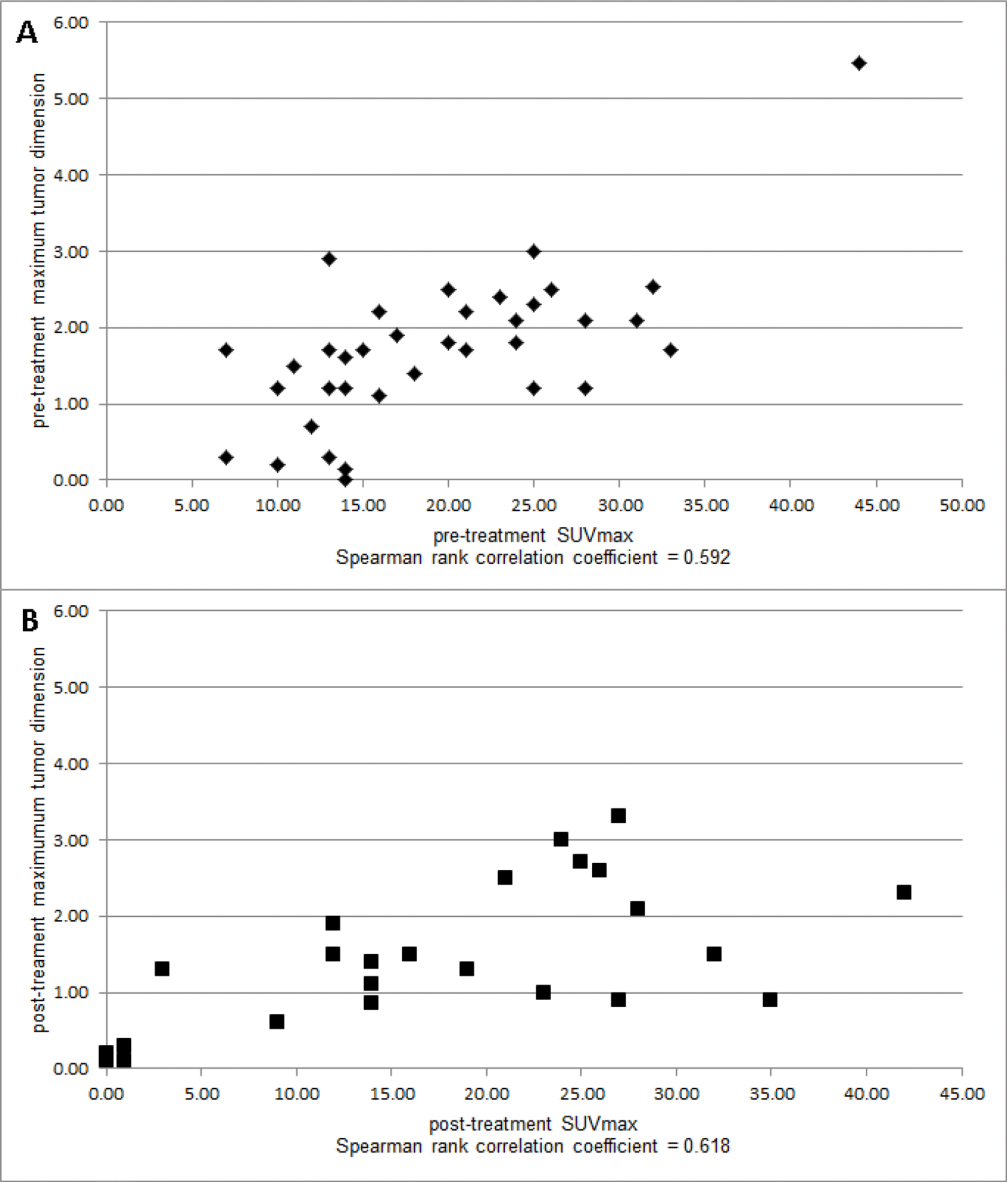
Correlations between tumor size (maximal diameter) and SUVmax of individual sites of tumor metastases pre-immunotherapy (**A**) and post-immunotherapy (**B**).

The number of metastatic lesions identified on pre-treatment ^99m^Tc-HYNIC-IL2 scans of these 5 patients ranged for 3 to 16 (median = 5; total number = 36). ^99m^Tc-HYNIC-IL2 scans showed a normal bio-distribution of the radiopharmaceutical at 1h images with clear disappearance from blood and concentration in liver and kidneys being the organs that normally metabolize IL2. Planar and SPECT images of neoplastic lesions showed the uptake of radiopharmaceutical to varying extents (Figures 1–5). The correlation between a lesion's maximum tumor dimension and its SUV_max_ prior to treatment was 0.592 (*n* = 36; *p* = 0.0001, Figure 6A). Among the 3 patients with a 12 week on treatment ^99m^Tc-HYNIC- IL2 scan, 22 of the 24 lesions seen on CT scan prior to treatment were still present at 12 weeks. The SUV_max_ in the area where the other two lesions were on their pre-treatment CT scan was 0.1 and 0.2 The correlation between maximum tumor dimension and SUV_max_ after 12 weeks on treatment was 0.618 (*n* = 24; *p* = 0.0013, Figure 6B), Moreover, the correlation between the percent change in SUV_max_ and the percent change in maximum tumor dimension was 0.654 (*n* = 24; *p* = 0.005) and the correlation between the change in SUV_max_ and the change in tumor dimension was 0.524 (*n* = 24; *p* = 0.0086). Results were similar when analyses were carried out using SUV_mean_.

Of the 3 patients with a 12 week ^99m^Tc-HYNIC-IL2 scan, one patient had a 91.1% reduction in tumor burden with a corresponding 71.8% reduction in total SUV_max_ from 7.1 to 2; a second patient had a 16.8% increase in tumor burden with a corresponding 6.3% reduction in total SUV_max_ from 29.3 to 27.45; and the third patient had a 9.1% increase in tumor burden with a corresponding 22.2% increase in total SUV_max_ 4.5 to 5.5.

### Histopathologic results

Due to the small number of patients, the histologic analysis of the biopsied lesions is only descriptive (Table 2). The tissue samples were analysed for presence of TIL by H&E, and immunostained for CD3 (detection of T cells). The biopsied tumor metastases that exhibited SUV signals between 1.7 and 2.6 revealed low to moderate TIL invasion with predominantly CD3+ T cells. Of note, patient #5 underwent a pre-IO therapy biopsy of a tumor lesion with an SUV value of 0.1 (no tracer uptake), with a tissue biopsy demonstrating diffuse TIL involvement throughout the sampled tumor tissue (predominantly CD3+ cells). This unexpected result may represent a lack of IL2 receptor expression of the TIL suggesting a state of inactivation, possibly explaining the growing tumor mass in the context of TIL. Unfortunately, patient #5 refused week 12 reimaging due to IO treatment related side effects.

**Table 2:** Summary of tissue analyses for TIL (CD3)

	Pre-IO therapy			Post-IO therapy		
Patient #	SUV_max_	TIL within TME (H&E)	TIL phenotype (IHC)	SUV_max_	TIL within TME (H&E)	TIL phenotype (IHC)
1	2.5	Necrotic tissue, diffuse TIL, +1	CD3	2.6	Diffuse, +1	CD3
2	1.7	Diffuse (+1) and some patches of +2	CD3	na	na	na
4	2.1	Diffuse, +2	CD3	2.1	Patchy and diffuse +2 with areas of +3	CD3
5	0.1	Diffuse, +4	CD3	na	na	na

Note: Patient #3's biopsied tumor lesion was not imaged; na = not available; TME = tumor microenvironment.

## DISCUSSION

At times, early tumor infiltration by CTL may manifest as pseudo-progression presenting as transient tumor enlargement followed by tumor regression [1]. Unfortunately, present clinical imaging methods are unable to discriminate pseudo-progression from a true progression. Over the past decades, our work has focused on the search of a reliable imaging probe for non-invasive *in vivo*

imaging of TIL [11–13]. To date our best results have been obtained using ^99m^Tc-IL2 labelling with HYNIC-NHS and tricine as co-ligands. In the current study, we demonstrated that ^99m^Tc-HYNIC-IL2 scintigraphy is a safe non-invasive clinical examination to assess the presence and changes in TIL in metastatic melanoma lesions in patients undergoing IO therapy with ipilimumab or pembrolizumab. The correlation between maximum tumor dimension seen on CT scan and ^99m^Tc-HYNIC-IL2 uptake was 0.592 (*p* = 0.0001) prior to treatment and was 0.618 (*p* = 0.0013) after treatment. These data suggest that the heavier the infiltration of TIL the larger the lesion.

Based on prior study, ^99m^Tc-HYNIC-IL2 uptake *in vivo* has been shown to reflect the presence of CD25+ activated T-lymphocytes and the findings of this study, we believe that our hypothesize that lesions that appear larger on CT scan after treatment with increased max SUV values are pseudo progression due to increased TIL infiltration should be further explored in a larger patient population. Moreover, the observation from patient #5 that the high CD3+ TIL content in the tumor biopsy did not reflect a “SUV” content may be that the level of ^99m^Tc-HYNIC-IL2 tumor uptake is reflective of a TIL population that lacks the high-affinity IL2 receptor (activated T cells) and is therefore inactive, potentially as a result of PD1-PDL1 interactions in the TME. Recent improvements in multiomic immunohistochemical analyses of TME will allow testing of this hypothesis in future studies.

To date, several other approaches have been described for imaging T-cells *in vivo*. Anti-CD3 monoclonal antibodies have been radiolabelled, but they showed either long plasma half-life or high grade toxicity [11, 14–15]. To bypass the long half-life of monoclonal antibodies, radiolabelled diabodies have been proposed as an alternative, but human studies have not been published yet [16]. Tavarè *et al*. have reported on the possibility to selectively image CD8+ cells in humans [17] whereas others have preferred to directly label T-cells *in vitro* with long half-life isotopes such as 111In for SPECT or 89Zr for PET [18] but this may result in a high radiation dose to labelled cells with possible damage and altered circulation and migration capacity *in vivo*. Recently a guanosine analogue ([18F]F- AraG) has been investigated as potential probe to image T cells *in vivo* by PET/CT [19] but preliminary human results were not encouraging. Other attempts have been reported using engineered human T- cells [20].

A different strategy to predict the efficacy of check point immunotherapy could be an evaluation of the *in vivo* lesion uptake of radiolabelled ipilimumab or pembrolizumab [21]. However, these antibodies have a long half-life and images should be taken 24–72 h after injection thus requiring the use of long half-life radioisotopes such as 89Zr with high radiation exposure to the patient. More promising seems the use of 64Cu to radiolabel antibodies [22]. Thus, until the availability of a better radiopharmaceutical, radiolabelled IL2 remains the most specific and suitable probe to *in vivo* image CD25+ T-cells.

Overall, the results of our pilot study have provided preliminary evidence of the safety and feasibility of ^99m^Tc- HYNIC-IL2 scintigraphy and have led to a number of hypotheses to be tested in future studies.

## MATERIALS AND METHODS

### Patients and methods

This study enrolled individuals ≥18 years of age with histologically confirmed stage IV malignant melanoma who were to begin treated with standard-of-care ipilimumab or pembrolizumab. Patients were required to have either: (1) two lesions in the same organ such that at least one of these two lesions was measurable by RECIST criteria using either IV contrast enhanced CT or CT component of PET/CT or (2) three lesions in different organs and at least one of these 3 lesions was measurable by RECIST criteria using either IV contrast enhanced CT or CT component of PET/CT. Additional eligibility criteria included: Eastern Cooperative Oncology Group (ECOG) performance status of 0, 1 or 2; adequate blood chemistries (absolute neutrophil count ≥1500/mm^3^; platelet count ≥50,000/mm^3^; hemoglobin >10g/dL; AST ≤3 × the institutional upper limit of normal (ULN); and Alkaline phosphatase ≤3 × ULN [up to 5× allowed for patients with liver metastases]); and a tumor accessible for biopsy. Individuals were excluded from participation if they had had discontinued radiation, surgery, or systemic therapy ≤21 days prior to registration, more than 2 prior systematic regimens in the metastatic setting; uncontrolled or current infection, failure to recover from the side effects of prior chemotherapy or surgery, or known allergy to ^99m^Tc-HYNIC-IL2 or its components.

Women of child-bearing potential also underwent a serum pregnancy test within 7 days of registration. Pregnant and nursing mothers were not allowed to enroll. The Mayo Clinic Institutional Review Board approved this study and informed consents were obtained from each patient prior to study entry.

Patients underwent physical examination, blood tests and disease evaluation within two weeks prior to study entry. Following registration but prior to the start of treatment, and at completion of induction therapy (approximately week 12, concurrent with first standard of care assessment of treatment response in asymptomatic patients), patients underwent blood draws, toxicity evaluations, and CT imaging followed by ^99m^Tc-HYNIC-IL2 SPECT/CT imaging and subsequent core needle biopsy of a clinically accessible lesion.

### Immunotherapeutic treatment

Patients who chose to receive ipilimumab for their metastatic melanoma were to follow standard-of-care clinical guidelines (FDA approved) for therapy and supportive care. That is, Ipilimumab (3 mg/kg) was administered by IV infusion over 90 minutes every 3 weeks for a total of 4 doses. If the full treatment course could not be completed within 16 weeks, the drug was to be permanently discontinued. IPI therapy toxicities and dose modifications were to be managed in accordance to practice standards. Similarly, patients who chosed to receive pembrolizumab for their metastatic melanoma were treated according to standard-of-care clinical guidelines. Pembrolizumab was administered at a dose of 2 mg/kg intravenously every 3 weeks until tumor progression or a maximum of 2 years. Pembrolizumab therapy toxicities were to be managed in accordance to practice standards.

### ^99m^Tc-HYNIC-IL2 preparation and imaging

Succinimidyl-6-hydrazinopyridine-3-carboxylate (HYNIC-NHS) was conjugated to the commercially obtained IL2 as previously described [9–10]. Briefly, HYNIC-IL2 (100 μg) was incubated with tricine, ^99m^TcO ^-^ (370–740 MBq) and SnCl_2_ for 10 min at room temperature. After labelling, ^99m^Tc-IL2 was purified by reverse-phase chromatography using Sep-Pak cartridges and diluted in 10 mL of 5% glucose with 0.1% human serum albumin (HAS) before injection. Ultrafiltration through a 0.22 μm filter was performed to assure sterility. Labelling efficiency was analyzed using reverse phase HPLC and ITLC-SG chromatographic strips (Pall Corporation) with 0.9% NaCl as the mobile phase. Bacterial endotoxin test (BET) with the gel clot method, as well as the sterility test was carried out as part of the quality control evaluation. Negative result of BET had to be secured before the radiolabeled drug product was released for administration to a patient. Scintigraphy was performed, before starting immunotherapy and approximately 12 weeks later. Images were acquired 1h after IV injection of the radiopharmaceutical using clinically available tomographic SPECT/CT (Philips Precedence). Both planar antero-posterior images and SPECT/CT images of the regions of interest (known for the presence of metastases) were acquired. Planar images were acquired with a 512 × 512 matrix up to 500.000 counts and SPECT images were acquired with a 3° step modality for 20 seconds per frame. After iterative reconstruction and attenuation corrections by CT scan, SPECT

images were qualitatively analyzed and the degree of ^99m^Tc-HYNIC-IL2 uptake was calculated in each lesion by SUV_max_ and SUV_mean_ measurement using commercially available software kindly provided by Hermes Medical Solutions ltd (Stockholm, Sweden; SUV = activity concentration in tumor/injected dose/body weight). Each detectable lesion was also scored for its size (CT trans-axial slides) by measuring the largest diameter, as per RECIST. If a patient developed a ≥ grade 2 hypersensitivity/allergic reaction no further ^99m^Tc-HYNIC-IL2 imaging was to be done.

### Study design

A pilot study was conducted to assess the feasibility and safety of ^99m^Tc-HYNIC-IL2 scintigraphy in patients with metastatic melanoma undergoing cancer immunotherapy therapy. 99mTc- HYNIC-IL2 scintigraphy limiting toxicities (SLT) were defined as: ≥ grade 2 allergic reaction; ≥ grade 3 anaphylaxis, ≥ grade 2 injection site reaction, or ≥ grade 3 non-hematologic toxicity (not attributed to immunotherapy treatment/progression or a co-morbid condition). A sample size of 10 patients was chosen. If none of these 10 patients develop a SLT, then we can say with 90% confidence that the true percentage of patients who will develop a SLT is less than 33%. A secondary aim was to examine changes in TIL invasion of the metastatic melanoma in patients undergoing therapy with immune checkpoint inhibitors. An exploratory aim was to gain preliminary data on whether changes in 99mTc- HYNIC-IL2 scintigraphy uptake correspond to changes in tumor size on CT imaging.

Tumor burden was defined as sum of the longest dimension of all the lesions present on CT scan Total SUV_max_ was defined as the sum of the SUV_max_ of these lesions Spearman rank correlation coefficients were used to assess the association between: a lesion's maximum tumor dimension and its SUV_max_ prior to treatment, maximum tumor dimension and SUV_max_ after 12 weeks on treatment, percent change in SUV_max_ and the percent change in maximum tumor dimension and the change in SUV_max_ and the change in tumor dimension. A *t*-test was used to assess whether the correlation coefficient is significantly different than zero. All analyses were carried out using SAS 9.4 software.

### Histopathologic analysis of tumor core biopsies

Core needle biopsies of clinically accessible tumor masses were performed after ^99m^Tc-HYNIC- IL2 imaging was completed. The biopsy material was then immediately fixed in formalin and processed to paraffin embedding (FFPE), per standard clinical guidelines. At the end of the study, all available tissues were stained by H&E (tissue identification and lymphocyte infiltration) and immunostained for CD3 using clinically validated IHC methodology. In brief, FFPE tissue blocks were sectioned at 5 μm. Deparaffinization and immunohistochemistry (IHC) staining were carried out per standard clinical practice guidelines on the Ventana Benchmark XT auto-staining (Ventana Medical Systems, Tucson, AZ) Slides were pretreated with Cell Conditioner 1 for 32 minutes followed by incubation with, 1:250 diluted anti-CD3, mouse anti-human monoclonal antibody (Clone LN10, Leica,

#NCL-L-CD3-565) at 37° C for 15 minutes. CD3 detection was done with OptiView DAB (Ventana Medical Systems). Normal tonsil was used as a positive control and normal tonsil without primary antibody used as a negative control. Level of lymphocyte tumor infiltration (TIL estimate) was performed using a semi-quantitative scoring scale: 0 = absent TIL, +1 = sparse/scattered TIL infiltrate (TIL comprising <1% visualized cells); +2 = mild TIL infiltrate, (TIL comprising 1–5% of visualized cells); +3 = moderate TIL infiltrate (TIL comprising 5–15% of all visualized cells); and +4 = marked TIL infiltrate (TIL comprising >15% of visualized cells).
